# Purification and characterization of a novel plant lectin from *Pinellia ternata* with antineoplastic activity

**DOI:** 10.1186/2193-1801-1-13

**Published:** 2012-08-16

**Authors:** Zhenyu Zuo, Handong Fan, Xue Wang, Wei Zhou, Lingling Li

**Affiliations:** 1 College of Chemical Engineering and Technology, Wuhan University of Science and Technology, Wuhan, 430081 China; 2 Institute of Microbiology, Jiangxi Academy of Sciences, Jiangxi, 330029 China

**Keywords:** Lectin, *Pinellia ternata*, Antineoplastic activity, Purification

## Abstract

A novel *Pinellia ternata* lectin was purified from the bulbs of a Chinese herb *Pinellia ternata* using a combination of hydrophobic chromatography and DEAE-ion exchange chromatography. The lectin was found to be a homodimer of 12093.3 Da subunits as determined by gel filtration and MS. Biochemical characterization of the lectin revealed the existence of a glycoprotein, which contains 3.22% neutral sugars. The N-terminal 10-amino acid sequence of the lectin, QGVNISGQVK, has not been reported for other lectins. The lectin had a special agglutinating activity with mouse erythrocytes at a minimum concentration of 8.0 ug/ml. The lectin was stable in the pH range of pH 5–12 and temperatures up to 80°C for 30 min. The results of MTT experiment showed that the lectin had significant effect towards tumor cells, the maximum inhibition of cell proliferation with Sarcoma 180, HeLa and K562 cell line were 85.2%, 74.6% and 59.4% respectively. Experimental therapy in vivo also showed that PTL apparently inhibited transplanted tumor in mice. Flow cytometric analysis demonstrated that PTL inhibited the proliferation of Sarcoma 180 in a time- and dose-dependent manner through inhibiting the transition of G_1_/S and subsequently inducing G_0_/G_1_ cell cycle arrest. Thus, *Pinellia ternata* lectin displays a high potential for antitumor activity.

## Background

Lectins are carbohydrate-binding proteins of non-immune origin with the ability to recognize special sugars existing on cell surfaces as a result of cell agglutination (Peumans and Van Damme [1998]). They are ubiquitous occurred in microorganisms, plants and animals and have attracted great interest due to their varied physiological roles in cell agglutination (Khan et al. [2007]), anti-tumor (Liu et al. [2009, 2010]), immunomodulatory (Rubinstein et al. [2004]), antifungal (Herre et al. [2004]) and antiviral effects (Wong and Ng [2005]). Plant lectins have been extensively used for the detection, segregation and description of glycoconjugates using their carbohydrate binding properties (Peumans and Van Damme [1998]). In recent years, lectins are used to trigger vesicular transport into or across epithelial cells and used in food security management and diagnosis of disease. Furthermore, lectin-mediated drugs have been acquired to target specific cells and some lectins with anti-proliferative properties were isolated and characterized from different parts of the plant like seeds (Lin and Ng [2008]), leaves (Park et al. [1997]) and roots (Yan et al. [2010]).

*Pinellia ternata* is a traditional Chinese medicine which was used for the treatment of insomnia, eclampsia and termination of pregnancy for hundreds of years under the name of Banxia. It has been studied by many researchers due to the important discovery in the clinical application that the aqueous extract of *Pinellia ternata* contributed to the treatment of cervical carcinoma by smearing on the afflicted part (Lin et al. [2003]). Much research (Luo et al. [2000]; Chen et al. [2003]; Wu et al. [2011]) has been done on indigenous compounds like polysaccharide, astragaloside, isoflavonoids, triterpene, saponins and various trace elements in *Pinellas* radix. However, not much information is available regarding the bioactive proteins from this herb. Some researchers insisted that sitosterol was the constituent; however, Sun et al. found that the total proteins obviously inhibited ovarian cancer cell lines but showed no toxicity to human umbilical cord blood hematopoietic progenitor’s *in vitro* (Sun et al. [1992]; Zhu et al. [1999]). Fu et al. also found that the 30% (NH_4_)_2_SO_4_ deposition part of total proteins from *Pinellia ternata* rhizome could significantly inhibit human hepatocellular carcinoma cell line Bel-7402 growth and induce its apoptosis (Fu et al. [2007]). Therefore, we could easily speculate that the lectin in total proteins of *Pinellia Ternata* may be one of the effective constituents with anti-tumor activity. But to date, there are no reports about the bioactive proteins from *Pinellia ternata* with anti-tumor activity.

A novel lectin with hemagglutinating activity was purified from the bulbs of *Pinellia Ternata* using a combination of hydrophobic chromatography and DEAE-ion exchange chromatography and characterized for its antineoplastic property in the present work.

## Results

### Purification of lectin

**Figure 1:**
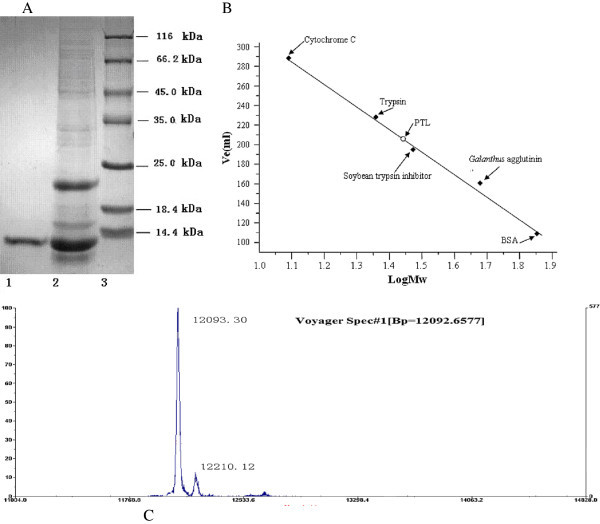
**Molecular mass determination.** (**A**): SDS-PAGE analysis. Lane 1, PTL purified by DEAE-sepharose chromatography. Lane 2, active fraction on PHE-sepharose chromatography. Lane 3, Molecular mass reference markers(beta-galactosidase (*E. coli*) (116.0 kDa), BSA(66.2 kDa), ovalbumin (45.0 kDa), lactate dehydrogenase (35.0 kDa), restriction endozyme (25.0 kDa), beta-lactoglobulin (18.4 kDa) and a-lactalbumin (14.4 kDa)). (**B**): Native molecular mass estimation of the lectin by gel filtration. Standard markers: BSA (67 kDa), *Galanthus nivalis* agglutinin (48 kDa), soybean trypsin inhibitor (30.2 kDa), trypsin (23.3 kDa) and cytochrome C (12.5 kDa). The molecular weight size markers (◆) and the lectin (○) are indicated. (**C**): Molecular mass determination by MALDI-TOF.

A lectin from *Pinellia ternata* was purified by a combination of ion exchange and hydrophobic chromatographic steps, which revealed strong agglutination activity with KM mouse erythrocytes. Hemagglutinating activity test was employed to monitor all the purification procedure. Fractionation of crude extract by precipitation with ammonium sulphate was dialysed against 20 mM Tris–HCl (pH 7.4) and loaded onto a PHE Sepharose Cl-4B column. Then active fraction was pooled and applied to DEAE-sepharose chromatography. A gradually enriched lectin with the agglutinating activity was purified and then was designated as *Pinellia ternata* lectin (PTL) (Figure 1A). A summary of its purification was provided in Table 1.

**Table 1:** **Specific hemagglutinating activities and yields of chromatographic fractions obtained at different steps of purification of lectin**

Fraction	Total protein(mg)	Specific hemagglutinating activity (HA/mg)	Total activity by HA	Purification fold	Recovery of protein (%)
Crude extract	2600	8.75	22750	1	100
hydrophobic chromatography	235	65.83	15470	7.5	9.04
ion-exchange chromatography	74	125.41	9280	14.3	2.85

Purification was initiated from 100 g of bulbs of *Pinellia ternata*, a lectin from *Pinellia ternata* was purified by a combination of ion exchange and hydrophobic chromatographic steps, which revealed strong agglutination activity with Kunming mouse erythrocytes. Hemagglutinating activity test was employed to monitor all the purification procedure. The total activity was measured by hemagglutinating activity assay as described in “materials and methods” section.

### Properties of purified lectin

The physical and biochemical properties of the lectin were investigated. The molecular mass of PTL was estimated to be 12.1 kDa using SDS-polyacrylamide gel electrophoresis (SDS-PAGE) (Figure 1A) or 25.8 kDa using gel filtration on the Sephacryl S-100 (Figure 1B). The mass spectrometry analysis appeared as a peak corresponding to m/z 12093.30 Da (Figure 1C). These results indicated that PTL is a homodimer consisting of two identical subunits of 12093.30 Da. Carbohydrate analysis using the phenol-sulfuric acid assay revealed that PTL was a glycoprotein with a neutral carbohydrate content of 3.22% (data not shown). Amino acid composition analysis of PTL indicated that Asp, Val, Glu, Ser, and Lys were present in higher concentrations than other amino acids (Table 2). The first 10 N-terminal amino acid sequence of PTL was determined as QGVNISGQVK.

**Table 2:** **Amino acid composition of the lectin from the bulbs of*Pinellia ternata***

Amino acid	Mol (%)	Amino acid	Mol (%)	Amino acid	Mol (%)
Asp	14.17	Val	12.50	Glu	11.67
Ser	10.00	Lys	8.33	Leu	6.67
Gly	5.83	Thr	5.00	Ala	5.00
Gln	4.17	His	3.33	Arg	3.33
Lle	2.50	Asn	1.67	Pro	1.67
Met	1.67	Cys-s	0.83	Phe	0.83
Tyr	0.83	Trp	0		

### Effect of temperature and pH

**Figure 2:**
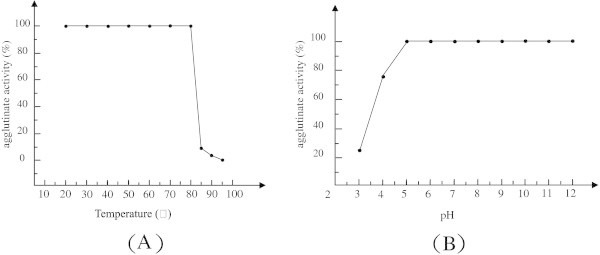
**Thermal stability and pH stability of PTL on the agglutinating activity were performed as described in “materials and methods” section.** (**A**): PTL was incubated at various temperatures for 30 min then rapidly cooled on ice. The residual hemagglutination activity was tested at room temperature. The hemagglutination activity of an untreated sample, tested at room temperature, represented 100% activity. (**B**): PTL was incubated at room temperature (24°C) in 20 mg/ml of different pH buffers ranging from pH 3 to 12. The titer value obtained at pH 8.0 represented 100% activity.

Thermal stability of PTL was determined in the temperature range from 20°C to 95°C. The results indicated that PTL was fairly stable up to 80°C for 30 min. However, the activity decreased significantly at higher temperatures and was totally inactivated when incubated at 95°C for 30 min (Figure 2A). The pH sensitivity profile of the lectin is shown in (Figure 2B). PTL exhibited broad pH optima between pH 5 and pH 12, but its activity was completely lost at pH below 3.

### The antitumor activity of PTL *in vitro*

**Figure 3:**
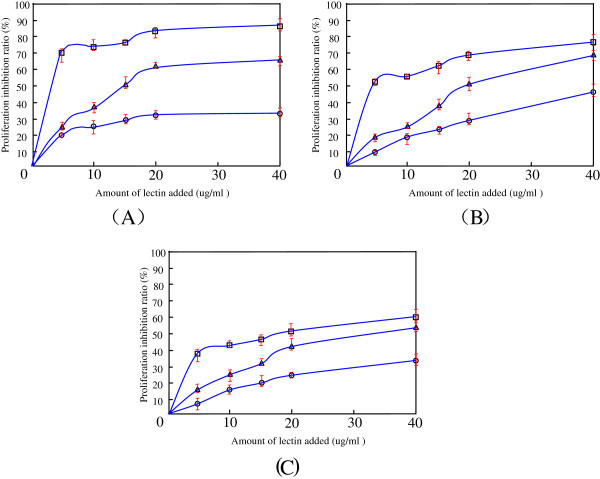
**Anti-proliferative effect of PTL on Sarcoma 180 cell line (A), HeLa cell line (B) and K562 cell line(C) were performed as described in “materials and methods” section**. Each data is expressed as the mean ± SD obtained from triplicate experiments. Symbols: 12 h (○), 24 h (▵), 48 h (□). Untreated cells were used for comparative purposes in this experiment and the Proliferation inhibition ratio was calculated according to the equation “materials and methods” section.

The anti-proliferative property of PTL against Sarcoma 180 cell line, Human cervical carcinoma cell line (HeLa) and human leukaemia K562 cell line are shown in (Figure 3). The maximum inhibitory effect of PTL on proliferation of Sarcoma 180, HeLa and K562 cell lines were observed with 40 μg/ml concentration for 48 h treatment. The maximum inhibition of cell proliferation with Sarcoma 180, HeLa and K562 cell lines were 85.2%, 74.6% and 59.4% respectively. Besides, the inhibition ratio for PTL in the above tested cell lines showed a concentration-dependence and time-dependence patterns. The results indicated that PTL showed maximum inhibition with Sarcoma 180 cells as compared to HeLa cells and K562 cells.

### The antitumor activity of PTL in vivo

PTL was intraperitoneal injected into Kunming mice with different concentrations and Cyclophosphamide (CTX) was used here as positive control to assess the antineoplastic effect of PTL on tumor growth. CTX is the most commonly used positive control in anti-cancer drug screening experiments and also the major chemotherapy drugs for cancer clinical treatment. Mice bearing Sarcoma 180 tumor exhibited a significantly (p<0.005) lighter tumor weight after PTL injection compared with the control mice, while animals injected with CTX showed a significantly lighter tumor body weight compared with the mice injected with PTL (Table 3). The inhibition rate were 15.6%, 32.1%, 36.2% according to the concentration of PTL (0.85 mg/kg, 2.30 mg/kg, 3.25 mg/kg) respectively. The results showed that PTL was an effective inhibitor toward tumor in vivo even though its effect was not as good as CTX.

**Table 3:** **Effect of PTL on growth of sarcoma 180 in mice**

	Dose ※ (mg/kg)	Tumor weight (g)	Inhibitory rate *(%)
CTX	20.00	0.463 ± 0.180	68.3 ± 12.2
PTL	3.25	0.935 ± 0.160	36.2 ± 10.9
	2.30	0.995 ± 0.131	32.1 ± 8.9
	0.85	1.236 ± 0.132	15.6 ± 9.0
Negative control (0.9%NaCl)	-	1.465 ± 0.122	-

The anti-tumoral effect was determined by examining the tumor weight and calculating the inhibition rate as described below. The value of P (p<0.005) was calculated according to the tumor weight at every experiment group.

※Each dose was tested in 12 mice every other day for 10 days;

*Inhibitory rate = [(tumor weight of control mice-tumor weight of experimental mice)/tumor weight of control mice]x100%.

### Flow cytometry (FCM) analysis

**Figure 4:**
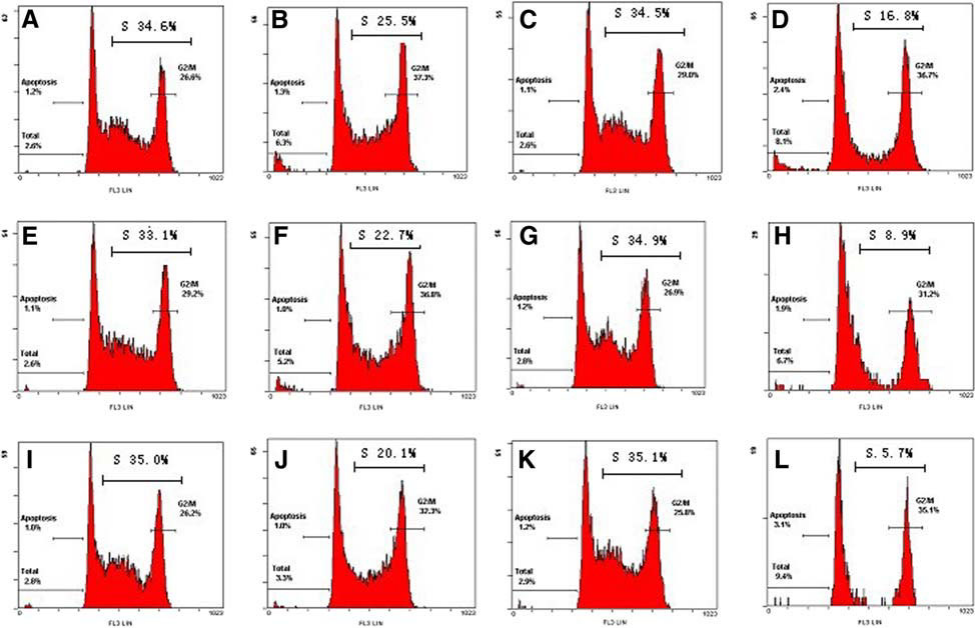
**Flow cytometry (FCM) analysis.** Sarcoma 180 cells were cultured with the methods described above and treated with different concentrations of PTL ranging from 0-40 μg/ml for 12 h and 24 h. For each analysis, a minimum of 10,000 cells were counted. A, E, I is the control for 12h and C, G, K is the control for 24 h, the incubating time is 12 h for B, F, J and 24 h for D, H, L. the concentration of PTL is 5 μg/ml for B and D, 20 μg/ml for F and H and 40 μg/m for J and L.

Aiming to elucidate the mechanism of inhibiting transplanted tumor in mice, the ability of PTL to inhibit cell cycle progression was analyzed by FCM. A representative example depicting the effect of PTL treatment for 12 h or 24 h on cell cycle phase distribution in the Sarcoma 180 cells is shown in (Figure 4). Treatment with 5, 20 and 40 μg/ml of PTL for 12 h or 24 h resulted in a significant decrease in the S DNA but little change in the G_2_/M DNA. As expected, no apoptosis was observed, which was in accordance with the results of MTT experiments. The decrease in the S DNA accompanied by the increase in the G_0_/G_1_ DNA was an indication of the inhibition of DNA replication, which might have taken place during the G_1_/S transition phase.

## Discussion

*Pinellia ternata* is one kind of traditional Chinese medicinal plant species, belonging to family Araceae. So far, majority of research reports concerning *Pinellia ternate* are around the aqueous extract and indigenous compounds of *Pinellia ternata*. Purification and characterization of one lectin with antitumor activity from *Pinellia ternata* have not been previously reported. Previous insect bioassay studies showed that *Pinellia ternata* agglutinin (PTA) from *P.ternata* had significant insecticidal activities. Yao et al. cloned the full-length cDNA of PTA with molecular weight about 29.4KD (Yao et al. [2003]). Wu et al. cloned another gene of *Pinellia ternata* agglutinin named as PTA-1, which encoded a lectin protein of 269 amino acids but had the similar molecular weight as PTA (Wu et al. [2010]). In the present study, PTL is a homodimer consisting of two identical subunits of 12093.30 Da, which is significantly different from PTA and PTA-1. In the present study, a novel lectin with obvious antitumor activity has been purified from *Pinellia ternata* bulbs for the first time using conventional chromatographic methods. The final yield of PTL is merely 2.85% after the procedure of hydrophobic chromatography and DEAE-ion exchange chromatography, approximately 740 mg lectin per kilogram dried material. PTL was found to be a homodimer with a molecular mass of 25.8 kDa. The lectin was a glycoprotein as detected by periodic staining. PTL was found to contain 3.22% of neutral sugars. In addition, PTL exhibited no sequence similarity with other previously reported lectins according to the search results from N-terminal homology, MALDI-TOF MS/MS analysis and database. Thus, PTL might be a novel araceae lectin, which has not been reported in any lectin family.

PTL, characterized by high thermostability and pH stability, exhibited agglutination towards Kunming mouse erythrocytes with the minimum agglutinating concentration of PTL 8 μg/ml. While lectin retained its full hemagglutinating activity up to 80°C for 30 min, PTL retained its full hemagglutinating activity in the broad pH range of pH 5 to 12. In addition, the activities of some other lectins are diminished above pH 9 reportedly (kaur et al. [2005]; Suseelan et al. [2007]; Vaz et al. [2010]).

Recently, the excellent antineoplastic activity of plant lectins has driven more and more attention to cancer studies (Jung et al. [2007]). Numerous studies have reported that some “ideal” anti-cancer candidate drugs can induce apoptosis in susceptible cancer cells (Nicholson [2000]). Like these anti-cancer drugs, PTL executed dose-dependent growth-inhibitory effect on three typical cancer cells. Sarcoma 180, HeLa and K562 cell lines showed growth-inhibitory responses to PTL by MTT assay. Moreover, further study about the anti-tumor activity of PTL in vivo has been performed in Kunming mice. The results showed that PTL apparently inhibited Sarcoma180 and transplanted tumor in mice, but the inhibition mechanism of PTL affecting proliferation and growth is still unknown. Is it PTL itself that transported to the tumor to initiate a biological response? Or does PTL in the intraperitoneal cavity cause the release of factors that via the blood are taken up by the tumor? Further in-depth research was needed to answer these questions.

In order to further investigate the mechanism of inhibiting transplanted tumor in mice by either inducing apoptosis or inhibiting cell proliferation, the effect of PTL on cell cycle progression was analyzed by FCM. The results suggested that PTL could cause a significant decrease of S phase in Sarcoma180, which indicated that the inhibition of cell growth was due to the arrest of DNA replication in the cell cycle. Thus the blockage effect of PTL occurred at G_1_/S transitions, correspondingly, the number of cells in G_1_/G_0_ phase increased and the number of cells in S phase decreased. In fact, a great number of studies have been reported that the arrest of cells at the checkpoints of the cell cycle occurs as an event happens preceding the detection of apoptotic cells (Balzarini et al. [2004]; Surh [1999]).

In summary, we purified PTL from The bulbs of *Pinellia ternata* and characterized some notable biological characteristics, especially its antineoplastic activity, for the first time. Due to accumulating evidence, the findings of PTL and many other plant lectins reported (Liu et al. [2009]; Yan et al. [2009]) would start a new exploration for plant lectins as potential anti-cancer candidate drugs due to their potentially remarkable antineoplastic activity. Furthermore, it might provide more promising insights into pharmaceutical exploitation in treatment of different human diseases in the near future.

## Materials and methods

### Materials

The *Pinellia ternata* purchased from Jinmen Hubei province had been identified for its authenticity by Keli Chen in Chinese Medicine of Hubei College of traditional Chinese Medicine; PHE Sepharose Cl-4B and DEAE Sepharose Fast Flow were purchased from Amersham Pharmacia Biotech; Roswell Park Memorial Institute 1640 (RPMI 1640) was from HyClone (USA); CTX was purchased from Wuhan University Zhongnan Hospital; MTT (3-(4,5-dimethylthiazol-2-yl)-2,5-diphenyltetrazolium bromide) and Dulbecco’s Modified Eagle’s Medium (DMEM) were products of Sino-American Biotechnology Company. Kunming Mice were purchased from Laboratory Animal Center of School of Medicine in Wuhan University and the experiments in vivo were performed in the same center. Mice blood was obtained by puncturing the marginal ear veins of healthy animals. The Sarcoma 180 and Hela cells were obtained from China Center for Typical Culture Collection. All other chemicals used were analytical grade reagents unless otherwise mentioned.

### Extraction and purification of lectin from the bulbs of *Pinellia ternata*

The bulbs of *Pinellia ternata* (100 g) were homogenized for 30 s at the internal interval of 1 min in deionized water at 4°C for 4 h. The extract was centrifuged at 8000 rpm for 40 min. The resultant pellet was discarded and the proteins in the supernatant were brought to 40% saturation of ammonium sulphate by stirring slowly, cooled at 4°C overnight and then centrifuged at 8000 rpm for 40 min. The precipitate was dissolved in 20 mM Tris–HCl buffer (pH 7.4) and dialyzed against the same buffer containing 1.5 M ammonium sulphate. After 16 h of dialysis, the sample was applied onto PHE Sepharose Cl-4B column which had been pre-equilibrated with “Tris–HCl buffer” mentioned above. Elution was carried out with the gradient buffer from “Tris–HCl buffer” containing 1.5 M ammonium sulphate to “Tris–HCl buffer” containing 0 M ammonium sulphate and monitored at 280 nm. The fractions exhibiting hemagglutinating activity were collected, dialysed against “Tris–HCl buffer” and then concentrated by centricon (PM-3). The concentrated active parts were loaded to a DEAE-Sepharose Fast Flow column (25 mm × 160 mm), which had been pre-equilibrated with “Tris–HCl buffer”. The bound protein was eluted by a continuous linear gradient of “Tris–HCl buffer” containing 0–0.3 M NaCl. The fractions exhibiting hemagglutinating activity were pooled and used for further studies. The purified lectin was designated as PTL.

### Protein concentration assay, hemagglutination assay and carbohydrate analysis

We measured the soluble protein content with the Bradford assay (Bradford [1976]) using bovine serum albumin as a standard. Hemagglutinating activity was measured in V-well microtitre plates (Cao et al. [2010]). A total volume of 0.1 ml was used in each well: 10 μl aliquots of serial three-fold dilutions of PTL in PBS, 20 μl of 2% suspension of mice erythrocytes in PBS and 70 μl of PBS were incubated for 2–3 h at room temperature and titre of visible agglutination by eye-sight was noted. One hemagglutination unit is defined as the lowest concentration of lectin that causes visible erythrocyte agglutination. The positive and the negative control were Concanavalin A and PBS respectively. Carbohydrate content of the purified lectin was determined by the phenol sulphuric acid method as described by the Dubois method using D-glucose as a standard (Dubois et al. [1956]).

### Homogeneity and molecular mass determination

SDS-PAGE was performed using 12.5% (w/v) acrylamide in gels, the molecular weight standard was the low molecular weight markers: beta-galactosidase (*E. coli*) (116.0 kDa), BSA (66.2 kDa), ovalbumin (45.0 kDa), lactate dehydrogenase (35.0 kDa), restriction endozyme (25.0 kDa), beta-lactoglobulin (18.4 kDa) and a-lactalbumin (14.4 kDa).

Native molecular mass of the purified lectin was detected by Sephacryl S-100 column (2.0 cm × 75 cm) which had been pre-equilibrated with 20 mM PBS (pH 7.4). Elution was carried out with the same buffer at a flow rate of 0.4 ml/min and monitored at 280 nm. The molecular mass of the eluting lectin was estimated from a plot of the log of the molecular weight, while a distribution coefficient (Kav) was calculated from the elution volume of the standard markers. The molecular mass standards used for calibration were BSA (66.2 kDa), *Galanthus nivalis* agglutinin (48 kDa), soybean trypsin inhibitor (30.2 kDa), trypsin (23.3 kDa) and cytochrome C (12.5 kDa).

Mass spectra were performed by using a Voyager-DE-STR MALDI-TOF mass spectrometer. The samples were pooled and redissolved in water containing 0.1% trifluoroacetic acid for desalting with C18 ziptips before being analyzed with MS.

### Amino acid composition and N-terminal determination

The lectin was hydrolyzed with 6 M HCl at 100°C for 30 h. The hydrolyzate residue in the supernatant was quickly derivatized with 9-fluorenylmethoxycarbonyl chloride and O-phthalaldehyde. The amino acid derivatives were analyzed by using HPLC with a Hypersil ODS C18 (4.6 mm × 150 mm) column. The N-terminal aa sequence analysis was performed by using an Applied Biosystems protein sequencer through automated Edman degradation.

### Temperature and pH profile

To study pH stability of the lectin, purified lectin was incubated at room temperature (24°C) in 20 mg/ml of different pH buffers ranging from pH 3 to 12: NaH_2_PO_4_-citric acid buffer (pH 3.0-5.5), sodium phosphate buffer (pH 6.0-7.5), Tris–HCl buffer (pH 8.0-9.0) and glycine-NaOH buffer (pH 9.5-12). After incubation for 30 min, the residual hemagglutinating activity was calculated.

To study the effect of temperature on hemagglutinating activity, purified lectin was incubated for 30 min at 20, 30, 40, 50, 60, 70, 80, 85, 90 and 95°C in PBS (pH 7.4). After incubation, aliquots were rapidly cooled on ice and the residual hemagglutinating activity was checked. The results were expressed as percentage of residual activity relative to the control.

### MTT colorimetric assay

Sarcoma 180 cell line, Human cervical carcinoma cell line (HeLa) and human leukaemia K562 cell line were used in the MTT colorimetric assay. Culturing and maintenance of these cells were followed as Yan (Yan et al. [2009]) and cell proliferation was checked by an MTT assay. Cells (1 × 10^5^) in their exponential growth phase were seeded into each well (200 μl media per well) of a 96 well plate and incubated at 37°C in a 5% CO_2_ incubator. After 12 h (the cell density was about 80%), cell culture were removed and new culture was added, different concentrations of PTL (pre-sterilised with 0.22 μm film filtration) ranging from 0–40 μg/ml were added to the well and the cells were further grown at 37°C for 48 h. After removing the supernatant, 200 μl of PBS containing 5 mg/ml MTT was added and incubated at 37°C for 4 h. The supernatant was removed again and 150 μl of dimethyl sulphoxide was added into each well to dissolve the MTT formazan at the bottom of the wells. After 10 min, the absorbance was read at 492 nm using enzyme-linked immunosorbent assay plate reader. The proliferation inhibition ratio was calculated using the following equation: Proliferation inhibition ratio (%) = [(U-T)/U] × 100%; where, U is the OD_492_ nm of the cells without PTL treatment (control) and T is the OD_492_ nm of the cells with PTL treatment. All experiments were carried out in triplicate.

### Establishment of sarcoma 180 tumors and experimental therapy in vivo

Sarcoma 180 cells were inoculated subcutaneously into male Kunming strain mice to establish tumors and this animal model of tumor was used to determine the antineoplastic effect of PTL on tumor growth. Kunming mouse has been widely used in medical experiments for its adaption to a variety of experimental environment. Sarcoma 180 cells, which were suspended in normal saline solution at 2.5 × 10^6^ cells/ml, were implanted subcutaneously on right front leg armpit (Kuznetsova et al. [1999]). Each mouse received 0.2 ml mixture of tumor suspension subcutaneously. After 24 h, PTL was maintained at the different concentrations ranging from 0–3.25 mg/kg body weight for intraperitoneal injection at every other day. CTX was given at 20 mg/kg body weight to the positive control group and 0.2 ml PBS solution was given to the negative control group. On the 11th day, after the mice were sacrificed, tumors and spleens indexes were extirpated and weighed. Later, the inhibition ratio of the tumors and the organ indexes were calculated as described by Chen (Chen et al. [2003]).

### Flow cytometry analysis

Sarcoma 180 cells were cultured with the methods described above and treated with different concentrations of PTL ranging from 0–40 μg/ml for 12 h and 24 h. The cells were processed by trypsin, collected and washed twice with ice-cold PBS, and then suspended in 75% ethanol at −20°C overnight. Fixed cells were centrifuged at 600 g for 5 min and washed with ice-cold PBS again. To study the DNA content and the cell cycle, cells were incubated with 100 μg/ml RNase A in PBS at 37°C for 1 h before stained in the dark with PI (20 μg/ml) at 4°C for 30 min. Samples were applied to a flow cytometer. For each analysis, a minimum of 10,000 cells were counted.

## Purpose of the work

Some researchers found that the total proteins of *Pinellia ternata* obviously inhibited ovarian cancer cell lines and we want to verify the lectin in total proteins of *Pinellia ternata* is one of the effective constituents with anti-tumor activity in this work.

## Abbreviations

PTL: Pinellia ternata lectin
SDS-PAGE: SDS-polyacrylamide gel electrophoresis
CTX: Cyclophosphamide
FCM: Flow cytometry
PTA: Pinellia ternata agglutinin
RPMI 1640: Roswell Park Memorial Institute 1640
DMEM: Dulbecco’s Modified Eagle’s Medium
MTT: 3-(4,5-dimethylthiazol-2-yl)-2,5-diphenyltetrazolium bromide.
